# Tuberculosis presenting as immune thrombocytopenic purpura

**DOI:** 10.1186/1476-0711-3-16

**Published:** 2004-09-06

**Authors:** Fahir Ozkalemkas, Ridvan Ali, Atilla Ozkan, Tulay Ozcelik, Vildan Ozkocaman, Esra Kunt-Uzaslan, Beril Bahadir-Erdogan, Halis Akalin

**Affiliations:** 1 Division of Hematology, Department of Internal Medicine, Uludag University School of Medicine, Uludag University Hospital, 16059, Bursa, Turkey; 2 Department of Chest and Tuberculosis, Uludag University School of Medicine, Uludag University Hospital, 16059, Bursa, Turkey; 3 Department of Microbiology and Infectious Diseases, Uludag University School of Medicine, Uludag University Hospital, 16059, Bursa, Turkey

**Keywords:** Tuberculosis, immune thrombocytopenic purpura, immune thrombocytopenia

## Abstract

**Background:**

Although various hematologic abnormalities are seen in tuberculosis, immune thrombocytopenic purpura is a rare event.

**Case Presentation:**

We report a case of a 29 year-old male who was presented with immune thrombocytopenia-induced hemoptysis, macroscopic hematuria and generalized petechiae. The patient was found to have clinical, microbiological and radiological evidence of active pulmonary tuberculosis. The immune thrombocytopenic purpura was successfully treated with anti-tuberculous drugs combined with corticosteroids and high dose immune globulin therapy.

**Conclusion:**

Immune thrombocytopenic purpura can be one of the hematological manifestations of tuberculosis which has a global prevalence with increasing incidence secondary to HIV infection.

## Background

During the past 2 decades, tuberculosis -both pulmonary and extrapulmonary- has re-emerged as a major health problem worldwide. Hematologic abnormalities have been described in association with mycobacterial infections for almost 100 years. Patients with both pulmonary and extrapulmonary tuberculosis (TB) may demonstrate peripheral blood abnormalities and findings may be minimal or profound [1,2]. A comprehensive review of the literature reveals a few case reports documenting tuberculosis as a cause of severe hematologic conditions such as hemophagocytic syndrome, thrombotic thrombocytopenic purpura and immune hemolytic anemia. Immune thrombocytopenic purpura (immune TP) associated with tuberculosis is exceedingly rare event. We report the case of immune TP associated with tuberculosis that was presented with severe hemorrhagic diathesis.

## Case Presentation

**Figure 1:**
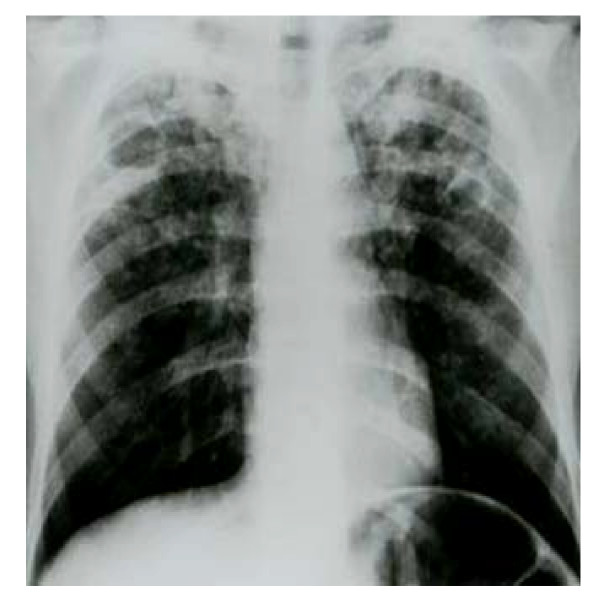
Anteroposterior chest radiograph showing cavitary lesions in both lungs

**Figure 2:**
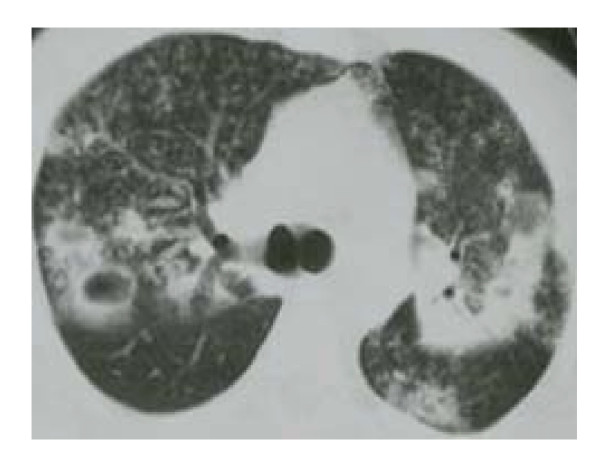
CT showing cavitary lesions in both lungs

**Figure 3:**
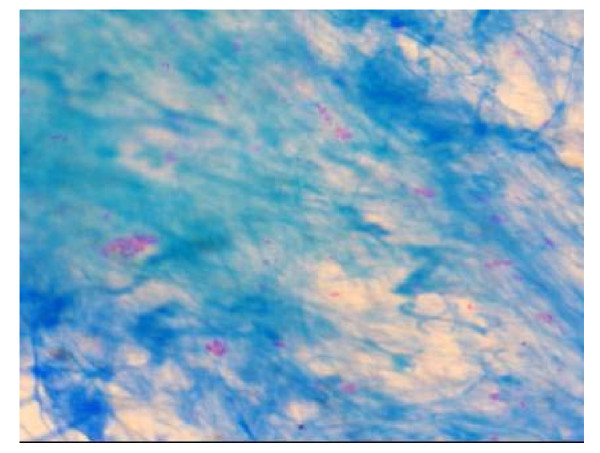
Sputum showing positivity of acid-fast bacilli

A 29-year-old previously healthy immigrant male patient from Kazakhstan was admitted to hospital with new-onset severe hemoptysis, macroscopic hematuria and extensive cutaneous petechiae on lower extremities. He appeared ill and poorly nourished. The patient was oriented and well cooperated, and there was no previous history of hematologic or liver or another disease and recent medication. He presented with unexplained weight loss of 2 months duration along with intermittent fever, night sweats and cough. The physical examination revealed a blood pressure of 100/70 mm/Hg, pulse 100/min, a temperature 37.2°C, extensive cutaneous petechiae on lower extremities, hemorrhagic bulla on tongue and on mucosa of oral cavity, and amphoric soufflé on apex of right chest. No organomegaly or lymphadenomegaly or evidence of another disease such as chronic liver disease was detectable. The initial complete blood count revealed a white blood cell 25.1 × 10^9^/l (58% neutrophils, 29% bants, 9% lymphocytes and 4% monocytes), hemoglobin 11.2 gr/dl, hematocrit 36%, MCV 84 fl, reticulocytes 1% and platelet count 7.6 × 10^9^/l. Erythrocyte sedimentation rate was 110 mm/h. A peripheral smear was remarkable for a paucity of platelets. Coagulation profile [prothrombin time (PT), activated-partial thromboplastin time (aPTT), fibrin degradation products (FDP)] were normal. A bone marrow aspiration demonstrated hypercellularity of all cell lines with normal maturation of myeloid and erythroid precursors. Megakaryocytes were increased in number with normal morphology. On bone marrow aspiration hemophagocytosis was not observed. A chest X-ray (Figure 1) and computed tomography (CT) (Figure 2) demonstrated bilateral patchy infiltrates and walled cavities on left and right upper lobes. Acid-fast bacilli were strongly positive in sputum (Figure 3). Bone marrow aspirate and urine for acid-fast bacilli were negative. Sputum culture yielded mycobacterium tuberculosis complex. The following laboratory studies were normal or negative: biochemical tests (glucose, urea, creatinine, uric acid, sodium, potassium, calcium, chloride, phosphorus, aspartate aminotransferase, alanine aminotransferase, alkaline phosphatase, lactate dehydrogenase, bilirubin, total protein and albumin), rheumatoid factor, anti-nuclear antibody, anti-platelet specific antibodies, Coomb's tests, HIV, hepatitis B and C virus, blood culture, bone marrow aspirate culture and abdominopelvic CT. No granuloma or hemophagocytosis was detected on bone marrow biopsy. The patient was started on rifampin 10 mg/kg/d, isoniazid 5 mg/kg/d, ethambutol 25 mg/kg/d, pyrazinamide 40 mg/kg/d, pyridoxine 75 mg/d and intravenous immune globulin (IVIg) 1 g/kg/d given for 2 days. On day 2 of hospitalization and treatment of anti-tuberculous therapy prednisolone 1 mg/kg/d was added. On day 8, platelet counts started to increase and on day 12 of the treatment it reached to 187 × 10^9^/l level. Patient improved on day 10 and he did not have any complaints on day 14; at time of discharge. He received a total of 6 red blood cell (RBC) units throughout hospitalization. During his hospitalization findings of hemolysis or gastrointestinal bleeding and massive bleeding in another site except hematuria and hemoptysis were not established. A complete blood count at discharge demonstrated a WBC 17 × 10^9^/l, Hb 9.6 g/dl, and platelet count 310 × 10^9^/l (Table 1). Corticosteroids were discontinued on day 14 of therapy and the patient was discharged and recurrent thrombocytopenia was not established after withdrawal of corticosteroid therapy. Ninety days after discharge, the patient was well with a platelet count of 300 × 10^9^/l (Table 1) and he had no side effect thought to be secondary to anti-tuberculous drugs.

**Table 1:** Characteristics of patient

Day	Hb (g/dl)	Hct (%)	WBC (×10^9^)	Plt (×10^9^)	Treatment
1	11.2	36	25.1	7.6	Anti-tbc drugs + IVIg
2	8	25	23.1	9.6	Anti-tbc drugs + IVIg+Pred+1 RBC Susp
3	8.1	25	22.7	17.7	Anti-tbc drugs+ Pred+ 2 RBC Susp
4	10.3	33	20.6	15.1	Anti-tbc drugs+ Pred
5	8.6	27	24.5	12.3	Anti-tbc drugs+ Pred+ 1 RBC Susp
6	8.2	24	25.4	4.0	Anti-tbc drugs + Pred + 2 RBC Susp
7	10.1	29	20.6	4.7	Anti-tbc drugs+ Pred
8	10.5	32	17.7	20.2	Anti-tbc drugs+ Pred
9	9.9	31	15.8	36.6	Anti-tbc drugs+ Pred
10	10.3	33	18	59	Anti-tbc drugs+ Pred
11	9.4	28	14	100	Anti-tbc drugs+ Pred
12	9.7	28	18.1	187	Anti-tbc drugs+ Pred
13	9.1	26	15.2	324	Anti-tbc drugs+ Pred
14	9.6	28	17	310	Anti-tbc drugs
45	12.3	37	15.5	304	Anti-tbc drugs
90	14	43	11	300	Anti-tbc drugs

Abbreviations: Anti-tbc drugs: anti-tuberculous drugs, Pred: Prednisolone, Susp: Suspension

## Discussion

Tuberculosis differs from many other infectious maladies in having particular social and geographic distributions. The disease was under control in developed nations and getting under control in developing nations, until the emergence of HIV infection and the advent of multidrug resistant strains of mycobacteria [3-5]. Various hematologic abnormalities such as anemia, leukocytosis, monocytosis, lymphopenia, leucopenia, thrombocytopenia, thrombocytosis, leukemoid reactions and pancytopenia have been seen in tuberculosis [1,2], but severe thrombocytopenia and presenting of tuberculosis as immune thrombocytopenic purpura is extremely rare and there are a few reports about tuberculosis induced immune thrombocytopenic purpura published in the world literature [6-10].

The case that we report could be confused by coincidental presentation of adult idiopathic thrombocytopenic purpura and tuberculosis, by drug-induced thrombocytopenia, thrombotic thrombocytopenic purpura (TTP)-hemolytic uremic syndrome (HUS), hemophagocytic syndrome and disseminated intravascular coagulation (DIC) associated with TB. Idiopathic thrombocytopenic purpura (ITP, also known as primary immune thrombocytopenic purpura) is an acquired disease of children and adults defined as isolated thrombocytopenia with no clinically apparent associated conditions or other causes of thrombocytopenia. Adult ITP typically has an insidious onset with long-lasting histories of purpura (thrombocytopenia for >6 months) and spontaneous remission is uncommon and is likely to be incomplete [11-13]. Steroids are the conventional first line therapy for adult ITP. Platelets counts increase within one week in responding patients and usually reach peak values by two to four weeks. However, in most patients, thrombocytopenia recurs when steroids are tapered or discontinued. Also in adult, IVIg is used when clinical situations require a transient increase of the platelet count and a typical response is an increase in platelet count several days after the infusions are initiated and return to the pretreatment level within several weeks [11,13]. In our case, we excluded the adult ITP not only by basing on standard criteria [12], but with response to steroids and IVIg therapy since thrombocytopenia did not recur after withdrawal of prednisone and IVIg therapy. Also we excluded other causes of thrombocytopenia such as hemophagocytic syndrome, TTP, combined autoimmune cytopenias with history, clinical and laboratory findings, and examination of bone marrow aspiration and biopsy that were described in case presentation.

Several factors are known to cause bleeding in association with infections, of which thrombocytopenia is the most common. The etiology of thrombocytopenia in most cases appears to be increased destruction of platelets such as due to DIC or septicemia without evidence of DIC or platelet adherence to damaged vascular surfaces or direct platelet toxicity caused by the microorganism or involvement of bone marrow. Adult acute immune thrombocytopenic purpura is defined as a bleeding disorder in otherwise healthy person caused by transient destruction of platelets. Although the most important therapy for infection-related thrombocytopenia is that directed at the underlying infection, treatment decisions for immune thrombocytopenic purpura remain controversial and may include single or combination therapy with corticosteroids, intravenous immunoglobulin (IVIg) according to degree of thrombocytopenia or hemorrhage [11,14]. The case that we reported was presented with symptoms of phthisis lasting for more than 2 months and severe hemoptysis, macroscopic hematuria and extensive cutaneous bleeding findings lasting for one week. Based on the clinical, radiological (X-ray and CT of chest) findings, demonstration of positivity of acid-fast bacilli in sputum and with exclusion of other causes of thrombocytopenia, immune thrombocytopenic purpura due to pulmonary tuberculosis was diagnosed. We applied anti-tuberculous therapy combined with corticosteroids and IVIg because of severe thrombocytopenia and severe hemorrhagic diathesis. Clinically, steroids are known to ameliorate the purpuric bleeding in patients before the platelet count actually increases. The early effect is due to decrease of vascular permeability. The effect of the steroids in the thrombocytopenia is probably complex and it is late effect. The mechanism of action of IVIg is unclear, but studies suggest blockage of the Fc receptors of the reticuloendothelial cells and suppression of antibody production and binding which may be a result of anti-idiotype antibodies that bind antiplatelet antibodies and modulate the immune response [14]. In our case, corticosteroids were discontinued on day 14 of therapy and the patient was discharged and recurrent thrombocytopenia was not established after withdrawal of corticosteroid therapy. These observations suggest that tuberculosis is the cause of thrombocytopenia in our patient. He received a total of 6 RBC units throughout hospitalization. He did not have findings of Coomb's positive or negative hemolytic anemia or microangiopathic hemolytic anemia, hemophagocytic syndrome, gastrointestinal bleeding and massive bleeding to thoracic cavity. But he had severe hemoptysis and macroscopic hematuria. Ninety days after discharge, the patient was in well health with a platelet count of 300 × 10^9^/l and he had no side effect thought to be secondary to anti-tuberculous drugs [15,16]. The patient is still in our follow-up without relapsing of thrombocytopenia.

In conclusion, since the incidence of tuberculosis is currently increasing in worldwide countries and it may present with different hematologic manifestations, in case of immune thrombocytopenic purpura tuberculosis should also be recalled. Finally, further studies are needed in order to fully characterize the pathophysiology and immunological abnormalities in tuberculosis-related immune thrombocytopenic purpura.
